# Reprogramming towards totipotency is greatly facilitated by synergistic effects of small molecules

**DOI:** 10.1242/bio.023473

**Published:** 2017-04-15

**Authors:** Kei Miyamoto, Yosuke Tajima, Koki Yoshida, Mami Oikawa, Rika Azuma, George E. Allen, Tomomi Tsujikawa, Tomomasa Tsukaguchi, Charles R. Bradshaw, Jerome Jullien, Kazuo Yamagata, Kazuya Matsumoto, Masayuki Anzai, Hiroshi Imai, John B. Gurdon, Masayasu Yamada

**Affiliations:** 1 Wellcome Trust/Cancer Research UK Gurdon Institute, University of Cambridge, Tennis Court Road, Cambridge CB2 1QN, UK; 2 Laboratory of Molecular Developmental Biology, Graduate School of Biology-Oriented Science and Technology, Kindai University, Wakayama 649-6493, Japan; 3 Laboratory of Reproductive Biology, Graduate School of Agriculture, Kyoto University, Kyoto 606-8502, Japan; 4 Institute of Advanced Technology, Kindai University, Wakayama 642-0017, Japan

**Keywords:** Nuclear transfer, Reprogramming, Epigenetic modification, Mouse

## Abstract

Animal cloning has been achieved in many species by transplanting differentiated cell nuclei to unfertilized oocytes. However, the low efficiencies of cloning have remained an unresolved issue. Here we find that the combination of two small molecules, trichostatin A (TSA) and vitamin C (VC), under culture condition with bovine serum albumin deionized by ion-exchange resins, dramatically improves the cloning efficiency in mice and 15% of cloned embryos develop to term by means of somatic cell nuclear transfer (SCNT). The improvement was not observed by adding the non-treated, rather than deionized, bovine serum. RNA-seq analyses of SCNT embryos at the two-cell stage revealed that the treatment with TSA and VC resulted in the upregulated expression of previously identified reprogramming-resistant genes. Moreover, the expression of early-embryo-specific retroelements was upregulated by the TSA and VC treatment. The enhanced gene expression was relevant to the VC-mediated reduction of histone H3 lysine 9 methylation in SCNT embryos. Our study thus shows a simply applicable method to greatly improve mouse cloning efficiency, and furthers our understanding of how somatic nuclei acquire totipotency.

## Abstract (precis)

**Summary:** The optimized culture condition with small molecules is sufficient to allow highly efficient mouse cloning by removing epigenetic barriers to reprogramming.

## INTRODUCTION

Nuclear reprogramming of a differentiated into undifferentiated embryonic cell state was originally demonstrated by the transfer of somatic cell nuclei into unfertilized eggs using frogs (Gurdon, 1962). Later, successfully cloned animals were reported in many mammalian species using similar strategies (Wakayama et al., 1998; Wilmut et al., 1997). These reprogramming technologies have been greatly accelerated by the finding of induced pluripotent stem (iPS) cells (Takahashi and Yamanaka, 2006). iPS cells are currently regarded as the most convincing source for regenerative medicine while mounting evidence suggests that somatic cell nuclear transfer (SCNT) into enucleated oocytes can result in embryonic stem cells with high quality from the cloned blastocysts (Kim et al., 2010; Ma et al., 2014; Tachibana et al., 2013). Moreover, nuclear transfer techniques offer unique opportunities to revive animals from frozen tissues, potentially including extinct animals (Folch et al., 2009; Wakayama et al., 2008), to produce transgenic animals from somatic cells and to propagate animals with beneficial traits. However, many of these promising attempts have been impeded by the low success rates of SCNT.

Tremendous efforts have been made to improve cloning efficiency. Since failures of epigenetic reprogramming have been often reported in cloned embryos during preimplantation development (Teperek and Miyamoto, 2013), small molecules to alter histone modifications are widely used to facilitate development of SCNT embryos (Ogura et al., 2013). The addition of trichostatin A (TSA), a histone deacetylase inhibitor, to culture medium of SCNT embryos for appropriate duration improves mouse cloning from less than 1% to 6.5% (Kishigami et al., 2006; Rybouchkin et al., 2006). TSA treatment increases levels of histone acetylation and reduces those of repressive histone methylation (Bui et al., 2010), which presumably results in correcting the aberrant expression of transcription factor genes in cloned two-cell embryos (Inoue et al., 2015). Recently, substantial improvements in mouse cloning have been shown by enzymatically removing repressive histone H3 lysine 9 trimethylation (H3K9me3) and up to 11% of SCNT embryos develop to term (Liu et al., 2016; Matoba et al., 2014). This is also true in the case of human somatic cell nuclear transfer (Chung et al., 2015). It is therefore desirable to develop a simple method to lower the level of H3K9me3 in SCNT embryos, for example by supplementation of small molecules to culture medium (Huang et al., 2016) in order to achieve dramatic improvement in animal cloning.

The effect of vitamin C (VC) on the reduction of histone H3K9 methylation and DNA methylation has been well documented in embryonic stem (ES) and iPS cells (Blaschke et al., 2013; Chen et al., 2013; Hore et al., 2016; Walter et al., 2016). VC mediates reduction of Fe^3+^ to Fe^2+^ (Hore et al., 2016), accelerating DNA and histone demethylation through the enhanced activity of Fe^2+^- and oxoglutarate-dependent enzymes (Monfort and Wutz, 2013). This role of VC as a potent epigenetic modifier, in addition to the well-characterized antioxidant role, allows enhanced reprogramming in iPS cells (Chen et al., 2013; Esteban et al., 2010; Stadtfeld et al., 2012; Wang et al., 2011). Reprogramming after SCNT is also aided by VC supplementation to embryo culture medium in mice (Mallol et al., 2015) and pig (Huang et al., 2011), although improvements in birth rates of cloned mice are modest compared to mRNA injection of histone modifiers (up to 5% versus 11%, respectively). The extent of epigenetic reprogramming and changes in gene expression need to be determined in VC-treated SCNT embryos. Another important aspect that has not been well explored is whether VC further enhances its effect on reprogramming in collaboration with other factors.

In this report, we examined synergistic effects of TSA and VC, two well-known epigenetic modifiers that can enhance reprogramming. We find that TSA and VC facilitate reprogramming at different times during the one-cell stage and, moreover, synergistically work for the development of SCNT embryos. Interestingly, the effect of TSA and VC is best exerted in our embryo culture condition containing deionized bovine serum albumin (dBSA) (Isaji et al., 2015), resulting in 15% of cloning efficiency. Mechanistically, expression of transcripts that are normally downregulated in cloned embryos is rescued by the sequential treatment of TSA and VC with dBSA. Our improved culture condition allows stable production of cloned mice, thus enabling the SCNT technology to be used for a variety of purposes.

## RESULTS

### The sequential treatment of trichostatin A and vitamin C boosts cloning efficiency in mice

**Fig. 1.**
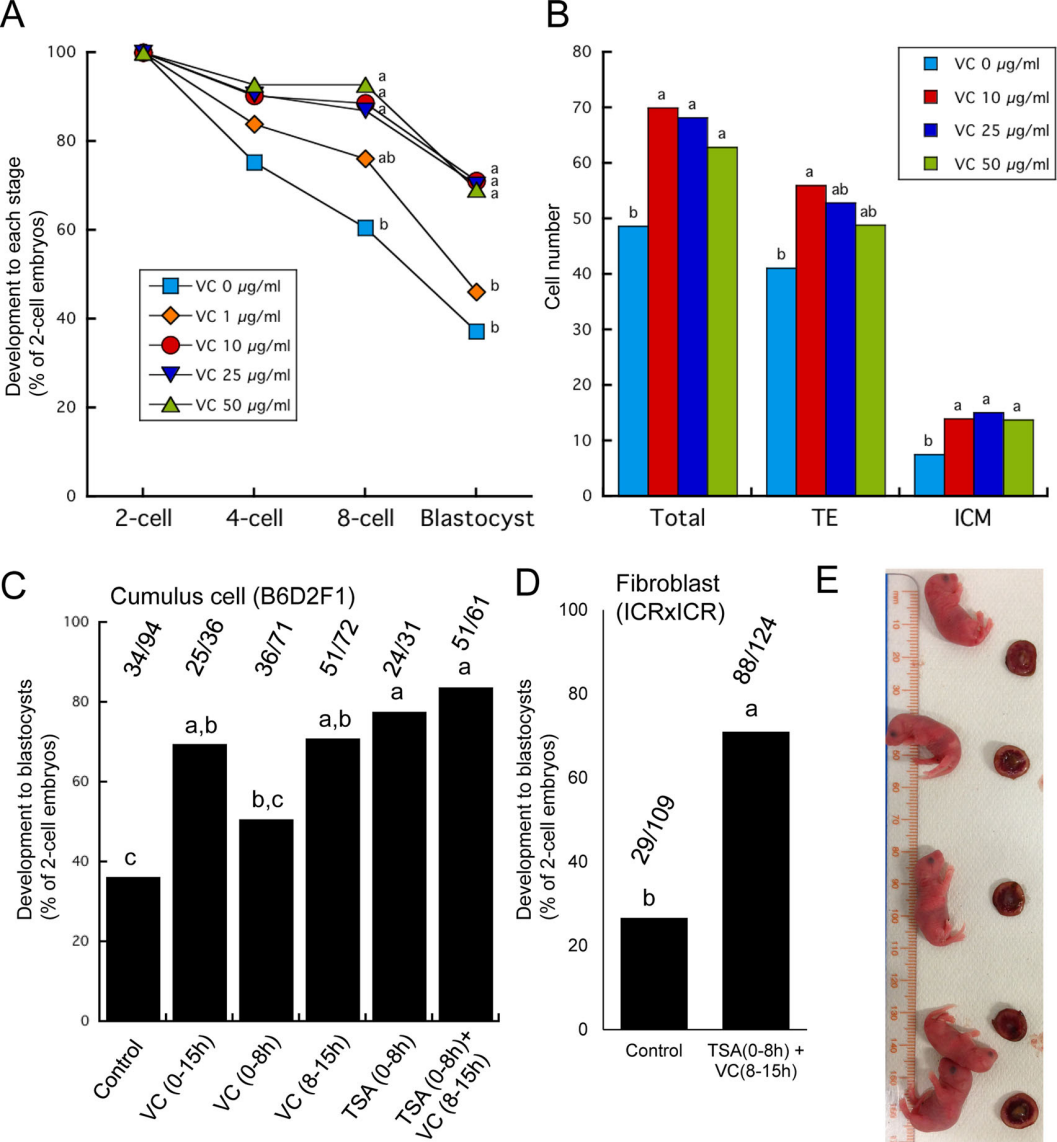
**The effect of epigenetic modifiers, trichostatin A (TSA) and vitamin C (VC), on the development of mouse SCNT embryos.** (A) The effect of different concentrations of VC on preimplantation development of the cloned embryos. VC at concentrations of 10 µg/ml and more enhanced development to the blastocyst stage. Different letters within the same stage indicate statistical significance (*P*<0.05, one-way ANOVA with subsequent Tukey's multiple comparison tests). Between 31 and 139 two-cell embryos were used in each treatment (each treatment: *n*=3-5, control VC 0 μg/ml: *n*=15). (B) Cell numbers of cloned blastocysts after the treatment with VC at concentrations of 10 µg/ml and more. Different letters within the same part indicate statistical significance (*P*<0.05). Between 12 and 31 blastocysts were examined (*n*=3-7). (C) Comparison of development to the blastocyst stage of the cloned embryos among VC treatment of different durations, TSA treatment and serial treatment with TSA and VC. Cumulus cells derived from B6D2F1 mouse were used as donor cells. Actual numbers of embryos developed to blastocysts per two-cell embryos are indicated above the corresponding graphs. Different letters indicate statistical significance (*P*<0.05; each treatment: *n*=3-6, control: *n*=12). (D) Comparison of development to the blastocyst stage of the cloned embryos using embryonic fibroblasts derived from ICR×ICR mice as donor cells between control non-treated embryos and serially treated embryos with TSA and VC. Actual numbers of embryos developed to blastocysts per two-cell embryos are indicated above the corresponding graphs. Different letters indicate statistical significance (*P*<0.05; *n*=4,6). (E) Five cloned fetuses and placentae in one foster mother, which were produced by the serial treatment of TSA and VC.

TSA has been shown to enhance the development of SCNT embryos when it is added to the medium for 10 h or less after egg activation, whereas longer exposure to TSA diminished enhanced embryonic development (Kishigami et al., 2006). We therefore hypothesized that treatment with VC also has a time window when its positive effects are maximized. As a first step, we tested the effect of different concentrations of VC on preimplantation development of mouse SCNT embryos using cumulus cells as donors. VC was supplemented in the embryo culture medium for 24 h after egg activation. Development to the blastocyst stage was significantly improved when 10 μg/ml or more of VC was supplemented (Fig. 1A). We then examined the number of cells at the blastocyst stage. Among the three different concentrations that enhanced development (10, 25 and 50 μg/ml; Fig. 1A), blastocysts treated with 10 μg/ml of VC exhibited a significantly higher number of inner cell mass and trophectoderm cells than control blastocysts (Fig. 1B; *P*<0.05). These results indicate that 10 μg/ml of VC enhances preimplantation development of mouse SCNT embryos. We next examined an appropriate timing of VC treatment for SCNT embryos. It has been reported that 15 h incubation with VC is enough to enhance the development of cloned embryos in pigs (Huang et al., 2011). We further split 15 h of treatment to 8 and 7 h [VC(0-8 h) and VC(8-15 h), respectively]. Interestingly, VC(0-8 h) did not enhance development to the blastocyst stage significantly, while the preimplantation development was improved by VC(8-15 h) (Fig. 1C). As previously reported, significant improvement of embryonic development by TSA was observed when it was added 0-8 h after premature chromosome condensation (Fig. 1C). Together, VC enhances preimplantation development of mouse SCNT embryos and its positive effect is observed when VC is added 8-15 h after egg activation, which represents a different timing from that of TSA treatment.

The optimization experiments of VC treatment for the development of SCNT embryos show that VC works at a different timing to TSA. We therefore hypothesized that the combinational treatment with TSA and VC might further enhance development of SCNT embryos. The sequential treatment with TSA and VC supported highly efficient *in vitro* development to the blastocyst stage [TSA(0-8 h)+VC(8-15 h); Fig. 1C]. Enhanced development was also observed using embryonic fibroblast cells derived from ICR×ICR mice (27% versus 71%; Fig. 1D), demonstrating that the positive effect of TSA and VC is neither limited by mouse strains nor by cell types. We then evaluated *in vivo* development of SCNT embryos with various controls using cumulus cells as donors (Table 1). VC treatment itself improved the implantation rate when SCNT embryos at the two-cell stage were transferred to surrogates (Table 1). An even higher implantation rate was obtained when TSA was supplemented before VC treatment [TSA(0-8 h)+VC(8-15 h); Table 1]. Importantly, birth rates were dramatically improved by TSA(0-8 h)+VC(8-15 h) treatment compared to all other treatments, including only TSA or VC treatment, and 15% of the transferred cloned embryos developed to live pups (Fig. 1E and Table 1). High birth rates of cloned pups were reproduced by three different operators in two different institutes (Fig. S1A,B). Cloned fetuses obtained by TSA(0-8 h)+VC(8-15 h) treatment showed similar body and placental weight to other treatments (Table 1). These results indicate that TSA and VC synergistically work to markedly improve the cloning efficiency in mice.

**Table 1.**
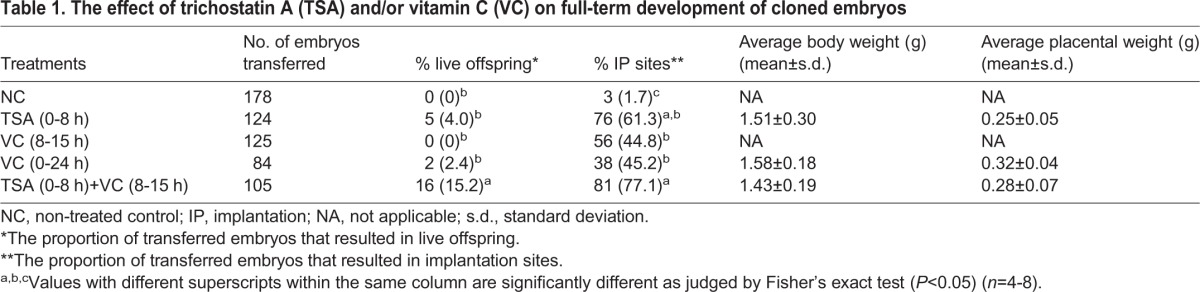
**The effect of trichostatin A (TSA) and/or vitamin C (VC) on full-term development of cloned embryos**

### The effects of antioxidants and deionized BSA on development of SCNT embryos

VC is known as an effective antioxidation reagent. We therefore asked if other antioxidants similarly enhance the development of mouse SCNT embryos. N-acetyl-L-cysteine (NAC) (Esteban et al., 2010) or vitamin E (Esteban et al., 2010) was added to the culture medium 8-15 h after the commencement of activation, which is the same timing as when VC's positive effect is observed. Although the VC treatment enhanced the development of SCNT embryos, neither NAC nor vitamin E improved the preimplantation development of mouse SCNT embryos (Fig. S2A). These results suggest that VC is not simply replaced by other antioxidants to enhance cloning efficiency. The improved development by VC treatment might not be through its antioxidant effects, although we cannot exclude some contribution of antioxidation to development.

Our culture medium contains deionized bovine serum albumin (dBSA), since commercially available BSA often involves trace amounts of transition metal ions, such as Zn^2+^ and Cu^2+^, harmful for preimplantation development (Vidal and Hidalgo, 1993). It is also known that VC is reactive to metal ions (Carr and Frei, 1999). We therefore tested if dBSA is crucial for the high developmental potential of TSA- and VC-treated embryos. Development to the blastocyst stage was impaired when BSA was supplemented in the medium and donor cell suspension instead of dBSA (Fig. S2B,C), suggesting that dBSA is also a critical factor for the improved cloning efficiency.

### Rescued gene expression in TSA- and VC-treated cloned embryos at the two-cell stage

**Fig. 2.**
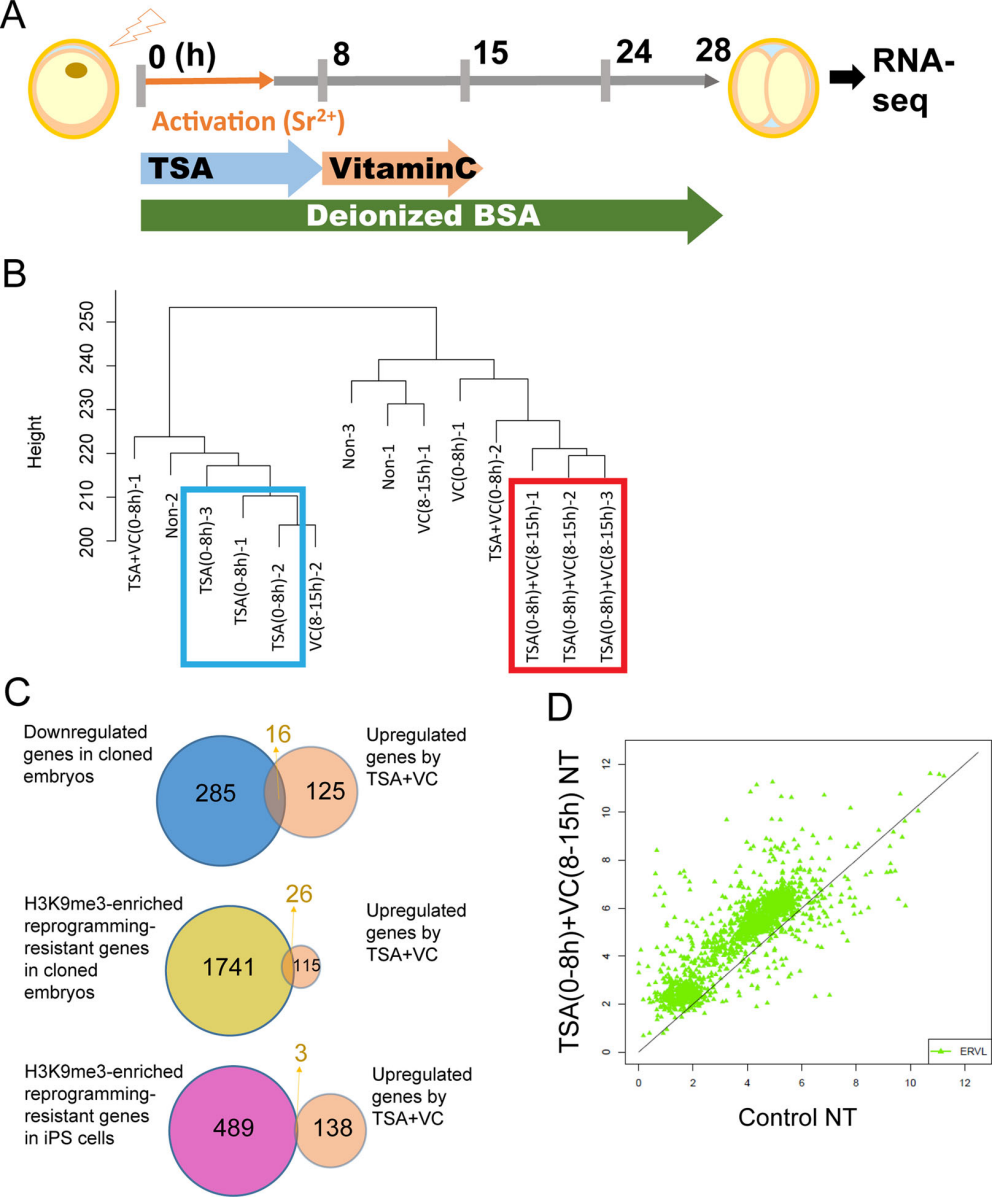
**RNA-seq analyses identify the reproducibly upregulated transcripts and retroelement in SCNT embryos incubated with TSA and VC in the medium containing deionized BSA.** (A) An experimental scheme for nuclear transfer with TSA and VC treatment, and subsequent RNA-seq analysis. (B) Hierarchical clustering analysis of the global gene expression profile after SCNT with various different treatments with TSA and/or VC. SCNT embryos incubated with TSA(0-8 h)+VC(8-15 h) are marked by the red square while those with TSA (0-8 h) are with the blue square. (C) Hypergeometric tests were performed among the lists of genes that were upregulated by TSA+VC, downregulated in cloned embryos (Matoba et al., 2014) and identified as reprogramming resistance in cloned embryos (Matoba et al., 2014) and in iPS cells (Soufi et al., 2012). (D) ERVL retroelement was upregulated by the treatment with TSA(0-8 h)+VC(8-15 h) when compared to the control non-treated SCNT embryos.

In order to understand the molecular basis of improved development of TSA- and VC-treated cloned embryos, we performed RNA-seq analyses. SCNT embryos at the two-cell stage (28 h after egg activation), which is right after zygotic genome activation, were used so that an immediate response of TSA(0-8 h)+VC(8-15 h) treatment to gene activation can be judged, and because a previous study identified abnormally expressed genes in SCNT embryos at the same stage (Matoba et al., 2014). Single SCNT embryos incubated in various conditions were subjected to RNA-seq (Fig. 2A). Hierarchical clustering indicated that the transcriptomes of SCNT embryos with TSA(0-8 h)+VC(8-15 h) treatment resembled each other even though nuclear transfer was performed in three independent experiments (Fig. 2B). Interestingly, the treatment with TSA(0-8 h) also resulted in a reproducible transcriptome, but it represented a significantly different pattern from TSA(0-8 h)+VC(8-15 h) (blue box versus red box; Fig. 2B). Heat map analysis showed reproducibly upregulated gene expression in SCNT embryos with TSA(0-8 h)+VC(8-15 h) (red box; Fig. S3A), compared to other treatments. Indeed, TSA(0-8 h)+VC(8-15 h)-treated SCNT embryos showed approximately two times more upregulated transcripts than downregulated transcripts when compared to control SCNT embryos (141 upregulated versus 63 downregulated transcripts, FDR<0.05; Fig. S3B and Table S1). GO analysis indicated that different categories of genes were found between up- and down-regulated transcripts (Fig. S3C). Upregulated transcripts included genes involved in transcriptional regulation and RNA processing (Fig. S3C), representing a similar category to transcripts previously identified as abnormally downregulated in cloned two-cell embryos (Matoba et al., 2014). We then asked if the transcripts upregulated in TSA(0-8 h)+VC(8-15 h) treatment contained the abnormally repressed genes in SCNT embryos (Matoba et al., 2014). Hypergeometric tests indicated that the abnormally downregulated genes were significantly enriched in our upregulated gene list by TSA(0-8 h)+VC(8-15 h) (top venn diagram, *P*=9.3×10^−13^; Fig. 2C), indicating that TSA+VC treatment at least partially rescues abnormal gene expression in SCNT embryos. Hypergeometric tests were also carried out against histone H3 lysine 9 trimethylation (H3K9me3)-enriched reprogramming-resistant genes identified in the mouse SCNT experiment (Matoba et al., 2014) and human iPS cells (Soufi et al., 2012). Reprogramming-resistant regions found in Matoba et al. (2014) contained 1767 genes, and 26 genes overlapped between those H3K9me3-enriched genes in SCNT embryos and the genes upregulated by TSA+VC (*P*=1.1×10^−6^; Fig. 2C; Table S2). In contrast, H3K9me3-enriched reprogramming-resistant genes identified in the iPS experiment did not show significant overlap with our gene list (*P*=0.44; Fig. 2C). In conclusion, abnormal gene expression of protein-coding genes, which is often observed in SCNT embryos, is alleviated by serial treatment with TSA and VC after nuclear transfer.

### TSA and VC differentially, but cooperatively, contribute to transcriptional activation in SCNT embryos at the two-cell stage

In order to gain insight into the synergistic effects of TSA(0-8 h)+VC(8-15 h), we sought to identify specific effects of TSA(0-8 h)+VC(8-15 h) on gene expression in SCNT embryos at the two-cell stage by comparing to other treatments such as TSA(0-8 h). In contrast to TSA(0-8 h)+VC(8-15 h)-treated SCNT embryos as described above (Table S1), TSA(0-8 h)-treated embryos showed approximately two times more downregulated transcripts than upregulated transcripts when compared to control SCNT embryos (43 upregulated versus 97 downregulated transcripts, FDR<0.05; Table S3); the detection of more downregulated transcripts is in good agreement with the previous study that also identified approximately two times more downregulated transcripts after the TSA treatment (Inoue et al., 2015). Nevertheless, among 43 upregulated transcripts, 13 transcripts were also found in the upregulated gene list in TSA(0-8 h)+VC(8-15 h)-treated embryos (Table S1), suggesting that at least some, but not all, transcripts upregulated by TSA(0-8 h)+VC(8-15 h) are due to the TSA treatment. We then examined the effect of the VC treatment by comparing TSA(0-8 h)-treated embryos to TSA(0-8 h)+VC(8-15 h)-treated ones. TSA(0-8 h)+VC(8-15 h)-treated embryos showed 3.7 times more upregulated transcripts than downregulated transcripts when compared to TSA(0-8 h)-treated embryos (153 upregulated versus 41 downregulated transcripts, FDR<0.05; Table S4). GO analysis showed that genes related to apoptosis were downregulated in TSA(0-8 h)+VC(8-15 h)-treated embryos. Genes related to apoptosis are known to be upregulated in SCNT embryos compared to fertilized embryos (Matoba et al., 2014), and the TSA(0-8 h)+VC(8-15 h) treatment, but not the TSA(0-8 h) treatment, downregulates such detrimental genes for embryonic development in SCNT embryos. In addition, 35 transcripts out of 153 upregulated transcripts (red highlighted in Table S4) are included in the 141 upregulated transcripts (Table S1), which were identified as differentially expressed genes between TSA(0-8 h)+VC(8-15 h) and control SCNT embryos. Furthermore, 13% of downregulated genes by the TSA(0-8 h) treatment (blue highlighted in Table S3; 13 out of 97 downregulated genes) were upregulated by the TSA(0-8 h)+VC(8-15 h). Taken together, the treatment with VC generally upregulates gene expression, including transcripts that are downregulated by the TSA treatment.

### Upregulated expression of the early-embryo-specific retroelement in SCNT embryos treated with TSA and VC

We next asked if treatment with TSA(0-8 h)+VC(8-15 h) affects the expression of retroelements, some of which are highly expressed in mouse embryos at the two-cell stage, including ERVL (Ishiuchi et al., 2015; Macfarlan et al., 2012). ERVL is suppressed in SCNT embryos, compared to IVF embryos (Matoba et al., 2014). Interestingly, the expression of ERVL was upregulated in the SCNT embryos treated with TSA(0-8 h)+VC(8-15 h), compared to the non-treated control SCNT embryos (Fig. 2D). Transcripts from LINE sequences were also upregulated and expression of other retroelements was not greatly affected (Fig. S4). These results suggest that the suppressed expression of the retroelement ERVL in the SCNT embryos is rescued by the TSA(0-8 h)+VC(8-15 h) treatment.

### Reduced histone H3K9 methylation is associated with a high developmental potential of TSA- and VC-treated SCNT embryos

**Fig. 3.**
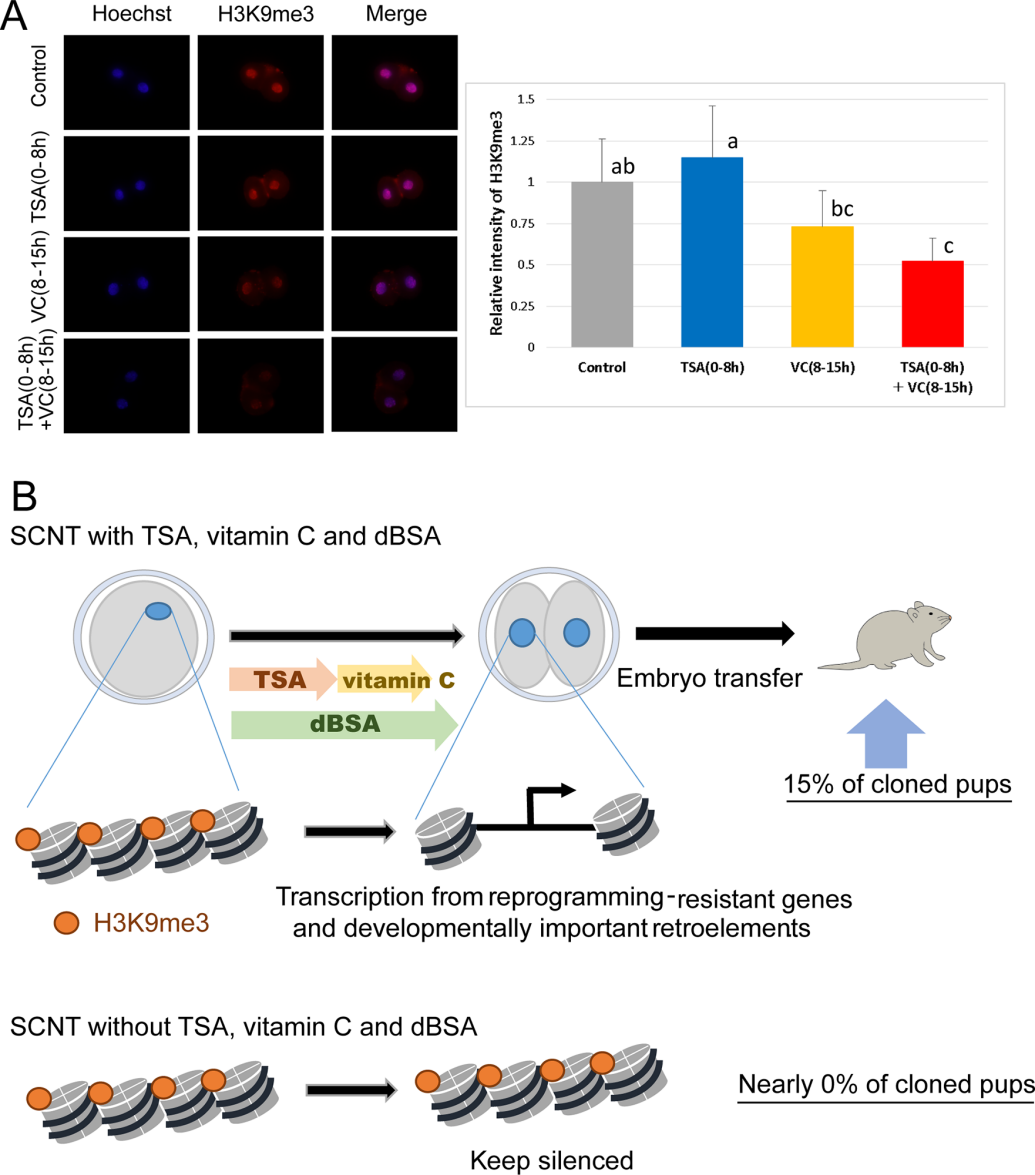
**Reduced histone H3K9 trimethylation in SCNT embryos treated with TSA and VC.** (A) Immunostaining analysis indicated that SCNT embryos with TSA+VC showed a significantly lower level of H3K9me3 compared to control embryos. Different letters indicate statistical significance (*P*<0.05, one-way ANOVA with subsequent Tukey's multiple comparison tests). Fourteen or fifteen two-cell embryos were examined. Error bars represent means±s.e. (*n*=3). (B) A model shows how the treatment with trichostatin A and vitamin C in the dBSA-containing medium enhances epigenetic reprogramming and results in the enhanced cloning success rate.

We have shown that TSA(0-8 h)+VC(8-15 h) treatment enhances transcription from H3K9me3-enriched reprogramming-resistant genes (Fig. 2C). Moreover, VC has been shown to accelerate epigenetic reprogramming including demethylation of DNA (Zhang et al., 2016) and histone H3K9 (Chen et al., 2013). We therefore examined changes in epigenetic modifications in TSA(0-8 h)+VC(8-15 h)-treated SCNT embryos. The upregulated expression of *Kdm3b*, a H3K9 demethylase, was observed in SCNT embryos after TSA(0-8 h)+VC(8-15 h) treatment (Fig. S5). A reduction in H3K9me3 was also observed at the two-cell stage after TSA(0-8 h)+VC(8-15 h) treatment (Fig. 3A). In addition, 5-hydroxymethylation (5hmC) was increased by TSA(0-8 h)+VC(8-15 h) treatment in SCNT embryos (Fig. S6) suggesting enhanced TET activities and DNA demethylation in SCNT embryos. Together, TSA(0-8 h)+VC(8-15 h) treatment decreases the abnormally high levels of H3K9me3 and DNA methylation in SCNT embryos, which may result in successful activation of reprogramming-resistant genes and the retrotransposons (Fig. 3B).

## DISCUSSION

Somatic cell nuclear transfer offers us a unique opportunity to experimentally render differentiated cells totipotent, enabling production of cloned animals from adult somatic cells. However, the adult somatic cells are resistant to this reprogramming and often retain somatic chromatin states even in reconstructed cloned embryos. As a result, SCNT embryos frequently exhibit aberrant histone modifications and gene expression, compared to normally fertilized counterparts. Landmark studies have discovered repeatedly observed abnormalities in SCNT embryos, including ectopic expression of noncoding RNA *Xist* (Inoue et al., 2010), repression of genes in large organized chromatin K9 modifications (LOCKs) (Fukuda et al., 2010; Inoue et al., 2010), defects in trophoblast cell lineage (Lin et al., 2011) and downregulated expression of genes enriched with H3K9me3 (Matoba et al., 2014). Previous studies have shown that correction of these representative defects greatly improves birth rates of SCNT embryos up to around 15% (Inoue et al., 2010; Lin et al., 2011; Liu et al., 2016; Matoba et al., 2014). The method we presented here also supports a similarly high birth rate of SCNT embryos (15.2%, Table 1). We achieved one of the most efficient mouse clones just by optimizing the culture condition without genetic manipulation, mRNA injection or help of tetraploid complementation. Thus, our simple method will be readily applicable to many laboratories and may be useful for cloning in other species, in which genetic manipulation, RNA injection and tetraploid complementation are not available.

Synergistic effects of histone deacetylase inhibitors with other factors to improve cloning efficiency have been explored because histone deacetylase inhibitors, including TSA, reliably enhance development of cloned embryos (Ogura et al., 2013). Despite numerous attempts, striking additive effects have not been observed (Inoue et al., 2010; Mallol et al., 2015; Matoba et al., 2014), apart from one of the highest mouse cloning rates to term achieved by the TSA treatment and siRNA injection against *Xist* (Matoba et al., 2011). We here show that sequential treatment of TSA and VC is key to maximizing their influences on reprogramming (Figs 1C and 2B, Table 1). TSA treatment, which begins right after nuclear transfer, increases histone acetylation in pronuclei and accelerates chromatin decondensation and proper organization of constitutive heterochromatin (Bui et al., 2010; Maalouf et al., 2009), suggesting that TSA contributes to initial remodeling of injected somatic nuclei presumably by relaxing chromatin. VC starts to enhance reprogramming 8 h after the commencement of activation, which represents approximately the same timing as the initiation of DNA replication and appearance of DNA repair markers, related to DNA demethylation (Wossidlo et al., 2010). In addition, this timing coincides with Tet3-mediated accumulation of 5hmC (Amouroux et al., 2016). Furthermore, demethylation of H3K9me2 can be induced until 10 h after activation (Wang et al., 2007). These results suggest that dynamic epigenetic reprogramming was induced in SCNT embryos when VC was added in our experiments. It is also noteworthy that the retroelement ERVL is transcribed as early as 8 h after oocyte activation (Kigami et al., 2003). Therefore, the proper erasure of somatic epigenetic memories and establishment of embryonic epigenetic signatures at this stage might be crucial for subsequent gene activation and hence embryonic development. In fact, our RNA-seq analyses revealed that both TSA and VC contribute to rescued gene expression in SCNT embryos at the two-cell stage, although the mechanism of action seems different between those two molecules. TSA induces not only activation of a set of genes but also downregulation of gene expression, while VC generally supports transcriptional activation including those downregulated by the TSA treatment.

The effect of TSA and VC was well-exerted when dBSA was added to mouse embryo culture medium instead of BSA. dBSA has been used as a component of culture media for human bone marrow cells (Leary et al., 1990) and for differentiating human ES cells (Ng et al., 2008). Injection of dBSA into oocyte cytoplasm enhances development of SCNT embryos while addition of dBSA to culture medium does not support better development than control (Isaji et al., 2015). This is in good agreement with our results that only dBSA did not support efficient development (non-treated control in Table 1). Nevertheless, the obvious requirement of dBSA for efficient development was seen when dBSA was used in combination with TSA and VC (Fig. S2), indicating synergistic effects of TSA, VC and dBSA. One possible explanation for this additive effect is that deionization of BSA removes metal ions that are reactive to VC from culture medium, thus increasing the ratio of free VC that can work with intracellular iron ion to increase the activities of Fe^2+^- and oxoglutarate-dependent enzymes to accelerate demethylation. Further investigation of dBSA may offer opportunities to reveal new mechanistic insight into reprogramming.

A key for efficient reprogramming is to break epigenetic barriers that stabilize somatic cell states. H3K9me3 has been convincingly shown as one of such barriers in SCNT embryos (Liu et al., 2016; Matoba et al., 2014) and in iPS cells (Chen et al., 2013; Soufi et al., 2012; Sridharan et al., 2013). Our study shows that the H3K9me3 barrier can be at least partially removed just by adding TSA and VC to the dBSA-containing medium at the appropriate timing (Figs 2C and 3B). H3K9 demethylation by VC is mediated through the elevated KDM3/4's activity in iPS cells, including KDM3B (Chen et al., 2013). Previous studies showed that the overexpression of *Kdm4b*, *Kdm4d* and *Kdm5b* enhances the development of mouse SCNT embryos (Liu et al., 2016; Matoba et al., 2014), while our TSA(0-8 h)+VC(8-15 h) treatment upregulates *Kdm3b* expression (Fig. S5). These results suggest that the improved development by the TSA(0-8 h)+VC(8-15 h) treatment might be mediated by a different mechanism from the previously observed effects of histone demethylase overexpression in SCNT embryos. One functional difference between *Kdm3b* and *Kdm4b/4d/5b* that has been shown to enhance mouse cloning is that *Kdm3b* demethylates H3K9me1/2 (Nottke et al., 2009), in good agreement with our observation that the H3K9me2-regualted retrotransposon MuERVL (Maksakova et al., 2013) is upregulated in SCNT embryos (Fig. 2D). Therefore, the improved development by the TSA(0-8 h)+VC(8-15 h) treatment might be not only through reducing H3K9me3, but also by downregulating H3K9me1/2. Moreover, hydroxymethylation was increased by TSA(0-8 h)+VC(8-15 h) treatment in SCNT embryos (Fig. S6), suggesting enhanced TET activities and DNA demethylation in SCNT embryos. Importantly, incomplete erasure of the somatic type of DNA methylation is also regarded as a major obstacle for successful reprogramming (Mikkelsen et al., 2008). These results demonstrate that TSA+VC treatment in SCNT embryos can overcome epigenetic barriers, resulting in the dramatic improvement of mouse cloning. Although TSA+VC treatment clearly affects epigenetic changes as mentioned above, several lines of evidence demonstrate that the development of SCNT embryos is also influenced by non-epigenetic factors (Miyamoto et al., 2011; Mizutani et al., 2012). For example, abnormal chromosome segregation is a major cause of developmental arrest of cloned embryos (Mizutani et al., 2012), which may in turn result in abnormal gene expression. Inhibition of class IIb HDACs, whose targets include not only histones but also chaperones and tubulin, plays a key role for improved development of cloned embryos (Ono et al., 2010). Furthermore, two-digit cloning rates can be achieved by using latrunculin A, an actin polymerization inhibitor, instead of cytochalasin B (Terashita et al., 2012). It is therefore plausible that the observed positive effects of TSA+VC treatment in the dBSA-containing medium might be accomplished by the combination of epigenetic and non-epigenetic factors, which warrants further investigation into the contribution of non-epigenetic factors. In conclusion, our refined protocol for mouse somatic cell nuclear transfer will ensure the efficient production of cloned mice and will make the SCNT technique widely available for a variety of applications, including preservation of genetic materials and regenerative medicine.

## MATERIALS AND METHODS

### Animals

B6D2F1 (C57BL/6J×DBA/2N) and ICR mice were obtained from Japan SLC (Hamamatsu, Japan) or CLEA Japan (Tokyo, Japan). This study conformed to the Guide for the Care and Use of Laboratory Animals. All animal experiments were approved and performed under the guidelines of the Animal Research Committee, Kyoto University and Kindai University, Japan.

### Collection of oocytes and cumulus cells

Female B6D2F1 mice, aged 8-10 weeks, were superovulated by intraperitoneal injections of 7.5 IU of pregnant mare serum gonadotropin (PMSG; ASKA Animal Health, Tokyo, Japan) followed 48 h later by 7.5 IU of human chorionic gonadotropin (hCG; ASKA Pharmaceutical, Tokyo, Japan). Cumulus–enclosed oocytes at the second meiotic metaphase (MII) stage were collected from the oviducts 15 h after the hCG injection in Hepes-buffered CZB medium (HCZB) (Chatot et al., 1989) and treated with 0.1% hyaluronidase in HCZB at 37°C until dispersion of cumulus cells. Cumulus-free oocytes were then washed and kept in drops of 0.3% dBSA (see below) containing KSOM medium without EDTA (mKSOM), covered with mineral oil, at 37°C in air containing 5% CO_2_ until use. Dispersed cumulus cells were removed from the hyaluronidase and placed in 6% dBSA containing HCZB until being used as donor cells for SCNT. For experiments shown in Fig. 1D, mouse embryonic fibroblast cells were derived from day 11.5 mouse embryos (ICR×ICR).

### Preparation of deionized bovine serum albumin

Stock solution of dBSA was prepared as described previously (Isaji et al., 2015; Ng et al., 2008). Briefly, BSA (Sigma-Aldrich, cat. code: A3311) was dissolved in distilled water at a concentration of 12%. Approximately 360 mg of mixed ion-exchange resin beads [AG501-X8(D); Bio-Rad] was then added to 10 ml of 12% BSA solution, and the mixture was incubated at room temperature with occasional stirring. When the beads changed color from blue-green to gold, fresh beads were replaced in the BSA solution for a total of three replacements. The supernatant was sterilized with filtration (0.45 µm, Millipore) and stored at −20°C as 12% stock solution. In mKSOM medium for culturing SCNT embryos, 0.3% BSA was supplemented. For Fig. S2B, BSA purchased from Calbiochem (San Diego, CA, USA, cat. code: 126609) was used to compare with the above mentioned dBSA.

### Somatic cell nuclear transfer

SCNT was carried out as described previously (Isaji et al., 2015). Briefly, enucleation of denuded MII oocytes was performed in drops of HCZB containing 5 µg/ml cytochalasin B (Sigma-Aldrich). After enucleation, a donor cell in HCZB with 6% dBSA was placed in the perivitelline space of an enucleated oocyte together with HVJ-E (GenomeONE-CF, Ishihara Sangyo, Osaka, Japan) by tightly attaching the donor cell to the enucleated oocyte, the oocyte was then cultured in mKSOM for 1 h at 37°C in air containing 5% CO_2_, during which time it fused with the donor cell. The reconstructed oocytes were activated by the incubation for 6 h in 5 mM SrCl_2_ and 2 mM EGTA-containing mKSOM supplemented with 5 µg/ml cytochalasin B, referred to as activation medium (Isaji et al., 2015; Kishigami and Wakayama, 2007), at 37°C in air containing 5% CO_2_.

### Epigenetic modifier and antioxidant treatments of cloned embryos

For epigenetic modifier treatments, trichostatin A (TSA, Sigma-Aldrich, cat. code: T8552) was dissolved in DMSO to prepare 10 µM stock solution and stored at −20°C. Vitamin C (VC, Sigma-Aldrich, cat. code: A5960) was dissolved in distilled water to prepare 1 mg/ml stock solution, filtered, and stored at −20°C. The final concentrations of both epigenetic modifiers were prepared by diluting the stock solution in activation or mKSOM culture media, depending on the experimental procedure (Figs 1 and 2A). The reconstructed oocytes were treated with 50 nM TSA for 8 h from the commencement of activation (COA), with VC at various concentrations (1, 10, 25 and 50 µg/ml) for 24 h after COA, with 10 µg/ml VC for 8 or 15 h after COA, or with 10 µg/ml VC during the period of 8 to 15 h after COA. The best developmental outcome was obtained by incubating the reconstructed oocytes with 50 nM TSA for 8 h after COA followed by treatment of 10 µg/ml VC for 7 h. For antioxidant treatments, N-acetyl-L-cysteine (NAC, Sigma-Aldrich, cat. code: A7250) and vitamin E (VE, Sigma-Aldrich, cat. code: T3634) were used. The reconstructed oocytes were treated with 1 mM NAC or 100 µM VE 8-15 h after COA. The activated oocytes were cultured at 37°C in air containing 5% CO_2_ in mKSOM before or after treatments with epigenetic modifiers or antioxidants until reaching the two-cell or blastocyst stages.

### Immunofluorescence staining

Cloned embryos at different developmental stages were fixed in 3.7% paraformaldehyde in PBS at 4°C overnight. After permeabilization with 0.5% Triton X-100 in PBS for 40 min at room temperature, samples were blocked in blocking solution (0.05% Tween-20, 1.5% BSA, and 0.5% sodium azide in PBS) for 1 h at room temperature. For 5meC and 5hmeC, the samples were treated with 4 M HCl for 10 min and neutralized with 100 mM Tris-HCl (pH 8.5) for 20 min. The samples were then incubated with primary antibodies at 4°C overnight. The primary antibodies used were rabbit anti-CDX2 (1:100, BioGenex, Fremont, CA, USA, cat. code: CDX2-88), mouse anti-trimethyl histone H3K9 (H3K9me3) (1:100, MBL, Nagoya, Japan, cat. code: MABI0318), mouse anti-5meC (1:200, Active Motif, Carlsbad, CA, USA, cat. code: 61479) and rabbit anti-5hmeC (1:200, Active Motif, cat. code: 39769) antibodies. After washing, the samples were incubated with the secondary antibodies, Alexa Fluor 488-conjugated anti-rabbit IgG antibody (1:500; Thermo Scientific, Waltham, MA, USA) or Alexa Fluor 594-conjugated anti-mouse IgG antibody (1:500; Thermo Scientific) for 1 h at room temperature. After another washing step, the samples were stained with 10 µg/ml Hoechst 33258 for 10 min and mounted on slides in 50% glycerol in PBS.

The fluorescence signals of CDX2, H3K9me3 and Hoechst were observed using a fluorescence microscope (FSX100, Olympus, Tokyo, Japan) using the same contrast, brightness, and exposure settings. Digital images of CDX2, H3K9me3 and Hoechst signals were acquired with the FSX-BSW software (Olympus). Confocal digital images of the fluorescence signals of 5mC and 5hmC were captured with a confocal laser-scanning microscope (Carl Zeiss, Germany) using the same contrast, brightness, and exposure settings. A semi-quantitative analysis of the fluorescence signals of H3K9me3, 5mC and 5hmC in the nuclei of each group was conducted using Image J. The total intensity of H3K9me3, 5mC and 5hmC in each nucleus was measured from five different random regions and the background value for the cytoplasm was subtracted. This calculated intensity was multiplied by the nuclear volume (v=4πr^3^/3) to yield the total amount of fluorescence for the nucleus (Bui et al., 2010).

### Embryo transfer

Pseudopregnant ICR females mated with sterile ICR males were used as embryo recipients. SCNT embryos that had developed to the two-cell stage were transferred to the oviducts of the pseudopregnant females at 0.5 days post-coitus (dpc). Cesarean section and uterine analysis of implantation sites were performed in all recipients at 19.5 dpc. If available, lactating foster mothers were used to raise live pups.

### Library preparation

Single embryos after the removal of zona pellucida were subjected to RNA purification using PicoPure RNA extraction kit (Thermo Scientific) according to the vendor's instruction. At the final step of RNA extraction, 9 μl of elution buffer was used to extract RNA from the column. Two μl of RNA out of 9 μl were subjected to Ovation Single Cell RNA-seq System (NuGEN) for making a library for sequencing. Two steps of PCR amplification (14 and 12 cycles) were needed to obtain an adequate amount of DNA for sequencing. The obtained library was quality-checked by TapeStation (Agilent) and quantified using Qubit (Thermo Scientific).

### Sequencing data filtering

Single end 50 bp sequencing was carried out using the Illumina Hiseq2000 system. Fastq files from Illumina sequencing were filtered for low quality reads (<Q20), and low quality bases were trimmed from the ends of the reads (<Q20). Adaptors were removed using cutadapt (Martin, 2011). The sequencing reads were then mapped to the mouse genome (mm9) using TopHat and UCSC annotation of exon locations in mm9. This annotation was also used to generate counts of reads covering exonic regions for each gene, and RPKM values were calculated from these by normalizing to total counts and transcript length.

### RNA-seq analysis

An unsupervised hierarchical clustering of the RPKM values was performed using hclust in R (using Euclidean distance and Ward agglomeration). To prepare the count data for differential expression analysis, a filter was applied requiring genes to have 1 count per million (CPMs) in all three treatment replicates or in all three controls. Differentially expressed genes were then identified using edgeR. Gene ontology terms showing over-representation of genes that were up- or down-regulated after nuclear transfer were detected using PANTHER (http://www.pantherdb.org/). Hypergeometric tests were performed to compare the overlap between upregulated genes in cloned embryos treated with TSA+VC and other published gene lists (Matoba et al., 2014; Soufi et al., 2012) (GSE59073 and GSE36570). Genes passing CPM filter in the control versus TSA+VC comparison were ordered by fold change. The RPKM values for these genes were z-score normalized across all samples and plotted on a heatmap.

RepeatMasker (http://www.repeatmasker.org/) was used to identify locations of retroelements in the mm9 genome. These were then used to prepare counts, CPMs and RPKMs for each retroelement and differential expression analysis was performed using edgeR (Robinson et al., 2010), all following the procedure detailed above. Scatterplots of RPKM values for TSA(0-8 h)+VC(8-15 h) NT and control NT were generated, splitting the retroelements by subtype.

### Statistical analysis

Percentage data were subjected to arcsine transformation before statistical analyses. Data on *in vitro* and *in vivo* embryonic development were analyzed by one-way ANOVA with subsequent Tukey's multiple comparison tests or Fisher's exact test. Comparison of blastocyst cell numbers and fluorescence intensity were performed by one-way ANOVA with subsequent Tukey's multiple comparison tests. A value of *P*≤0.05 was considered significant.
